# Is There an Increased Risk for Ischemic Stroke in Patients with Multiple Sclerosis, and If So, Should Preventive Treatment Be Considered?

**DOI:** 10.3389/fneur.2016.00128

**Published:** 2016-08-11

**Authors:** Steven M. LeVine

**Affiliations:** ^1^Department of Molecular and Integrative Physiology, University of Kansas Medical Center, Kansas City, KS, USA

**Keywords:** antiphospholipid antibodies, autoimmune, blood brain barrier, cerebral blood flow, cerebrovascular disease, multiple sclerosis, stroke, thrombosis

Multiple sclerosis (MS) is considered to be an autoimmune demyelinating disorder of the central nervous system (CNS), which leads to disruptions in sensory, motor, and/or cognitive systems. In addition to these disruptions, patients with MS have an elevated risk for various comorbidities. In order to provide comprehensive patient care, a proper understanding of these comorbidities is required.

The risks for various cardiovascular diseases (CVDs) have been examined in MS patients [reviewed in Ref. (1)], and ischemic stroke is one of the CVDs. Although an increased risk for ischemic stroke has been described for patients with MS, issues have been raised to question whether the observed elevated risk is valid. Since individuals who have an elevated risk for ischemic stroke are often given preventive treatment, clarifying whether MS patients have an actual elevated risk for ischemic stroke is an important first step in determining whether these patients, or a subgroup of patients, should be placed on a preventive treatment regimen.

A study by Roshanisefat et al. (2) determined that the relative risk for ischemic stroke was increased in patients with MS during the initial year of diagnosis, with statistical significance being lost if data from the initial period was removed from the analysis. Other studies (3, 4) also observed an elevated risk for ischemic stroke (or unspecified stroke) during the first year of diagnosis, although unlike the findings by Roshanisefat et al. (2), the increased risk persisted over the long term, albeit less than for the initial year. Additional studies, likewise, found a heightened risk for stroke in MS patients [reviewed in Ref. (1)], and multiple reports have described cerebral venous thrombosis in MS patients (5–8).

Roshanisefat et al. (2) provided the reasonable explanation that surveillance bias (e.g., an increased frequency of neuroimaging during the initial period) was probably responsible for the apparent elevated risk during the first year. In addition, Roshanisefat et al. (2) discussed that misdiagnosis of lesions could have contributed to the observed elevated risk, and the possibility of misclassification was raised by Allen et al. (9). These reasons were echoed by us in a review (10), and published reports describe examples of diagnostic challenges between MS and stroke (11–13). Together, these explanations questioned the accuracy of the findings of an increased risk for stroke in patients with MS. However, weighing of additional factors indicates that the increased risk might be valid.

The loss of a statistically significant increased risk of stroke in MS patients after excluding the initial year (2) could be accounted for by two mechanisms. First, it is possible that once a patient is diagnosed with MS, then stroke is underdiagnosed due to attributing findings consistent with stroke to the MS disease itself. The possibility that stroke was misdiagnosed as a MS flare during early years, e.g., 1977–1996, was put forward by Christiansen et al. (3). Second, the implementation of a disease-modifying therapy (DMT) would lessen inflammatory events, and thereby possibly lessen the occurrence of stroke. This might also explain why some other comorbidities also display a decreased relative risk after the initial year of diagnosis, e.g., pulmonary embolism across a wide range of autoimmune disorders (14).

It turns out that multiple autoimmune diseases have an increased risk for ischemic stroke. For example, besides MS or vasculitis, bullous pemphigoid and rheumatoid arthritis are associated with an increased risk for stroke (15, 16). The occurrence of antiphospholipid antibodies is also associated with stroke. Patients with antiphospholipid syndrome have autoantibodies that target phospholipids leading to an increased risk of vascular thrombosis with stroke being among the more common thrombotic events (17). The presence of antiphospholipid antibodies to β2glycoprotein I is associated with an increased risk of stroke in patients with systemic lupus erythematosus with neuropsychiatric manifestations (18). Furthermore, antiphospholipid antibodies may be associated with stroke in patients with Sjögren’s syndrome (19). Antiphospholipid antibodies are more common in patients with MS than in healthy control subjects, and they appear to be more prevalent during relapse (20, 21). Thus, it is possible that antiphospholipid antibodies contribute to the generation of stroke in MS patients, which could account for, at least in part, the elevated risk of ischemic stroke in this patient population. Additionally, extensive cerebrovascular changes (e.g., platelet activation and hypoperfusion) occur in MS, and these changes could promote clot formation in the CNS (10, 22). Other factors that could contribute to the elevated risk of stroke in patients with MS are the increased prevalence of smoking and reduced physical activity in this patient population (1).

After evaluating the body of data listed above, in my view, surveillance bias and/or misdiagnosis do not fully account for the observed elevated risk for ischemic stroke in patients with MS. Instead, pathogenic mechanisms, some of which appear to be shared across multiple autoimmune diseases, are likely responsible, at least in part, for the greater risk of stroke in patients with MS. If correct, then this raises the issue of preventive treatment.

There have been recent suggestions for pursing preventive measures directed against ischemic stroke in MS patients (4, 23). However, before considering a broad advisement for a preventive treatment regimen for patients with MS, in my view, two main questions should be addressed. First, do all MS patients have an elevated risk for ischemic stroke or is the risk more, or predominantly, pronounced within a subgroup(s) of patients? Some studies observed an increased risk for young patients (3, 4) while another study observed a suggestion for greater trend in older patients (23). It would not be surprising if different disease courses of MS (clinically isolated syndrome, relapsing remitting MS, primary progressive MS, and secondary progressive MS) or different states of disease activity have different associated risks for ischemic stroke. Patients who have restricted mobility, who smoke, or who have antiphospholipid antibodies could represent a subgroup having a particularly high risk (10). Also, it is possible that a DMT is contributing to (23), or reducing, an elevated risk.

Obtaining clear and complete answers about the risks in subgroups of patients likely would be a difficult and prolonged task. However, one possibility would be to screen MS patients for known risk factors for ischemic stroke as these may produce a compounded risk (e.g., the risk due to MS itself plus the risk due to the additional risk factor for ischemic stroke). Furthermore, it is possible that these known factors actually account for the majority of the elevated risk in MS patients. Thus, patients with known risks for ischemic stroke may be more favorable candidates for preventive treatment (possible greater benefit-to-risk ratio for treatment); however, this brings us to the second question: does the pathogenesis of MS present a particular susceptibility for an adverse event in response to an intervention? For instance, low dose aspirin is often used as a preventive treatment for individuals with an increased risk for ischemic stroke, but given that disruption to the blood–brain barrier (BBB) is a common pathological feature in MS, and aspirin increases the risk for hemorrhagic stroke and other bleeding events, it raises the question of whether aspirin could worsen BBB leakage (10). Given the importance of this potential issue, it is an area of investigation that I am interested in exploring in future research endeavors. I also encourage others to address the issues raised above. Once obtained, this information should be considered in the broader context of other comorbidities, so that a comprehensive picture can emerge about preventive treatment for patients with MS.

In summary, although the comorbidity of ischemic stroke for patients with MS is likely valid, I believe that there are outstanding issues that should be considered before applying preventive treatment strategies for MS patients in a broad-based manner. These issues include making sure that a preventive treatment does not have adverse effects that are particular to this patient population and determining which subgroups of patients most likely will benefit from preventive treatment.

## Author Contributions

The author confirms being the sole contributor of this work and approved it for publication.

## Conflict of Interest Statement

SL has received past and current funding from ApoPharma, Inc.
